# Prognostic Significance of CD26 in Patients with Colorectal Cancer

**DOI:** 10.1371/journal.pone.0098582

**Published:** 2014-05-28

**Authors:** Colin Siu-Chi Lam, Alvin Ho-Kwan Cheung, Sunny Kit-Man Wong, Timothy Ming-Hun Wan, Lui Ng, Ariel Ka-Man Chow, Nathan Shiu-Man Cheng, Ryan Chung-Hei Pak, Hung-Sing Li, Johnny Hon-Wai Man, Thomas Chung-Cheung Yau, Oswens Siu-Hung Lo, Jensen Tung-Chung Poon, Roberta Wen-Chi Pang, Wai Lun Law

**Affiliations:** 1 Department of Surgery, Li Ka Shing Faculty of Medicine, The University of Hong Kong, Hong Kong SAR, China; 2 Department of Clinical Oncology, Li Ka Shing Faculty of Medicine, The University of Hong Kong, Hong Kong SAR, China; 3 Centre for Cancer Research, Li Ka Shing Faculty of Medicine, The University of Hong Kong, Hong Kong SAR, China; Sapporo Medical University, Japan

## Abstract

**Background:**

CD26, dipeptidyl peptidase IV, was discovered firstly as a membrane-associated peptidase on the surface of leukocyte. We previously demonstrated that a subpopulation of CD26^+^ cells were associated with the development of distant metastasis, enhanced invasiveness and chemoresistance in colorectal cancer (CRC). In order to understand the clinical impact of CD26, the expression was investigated in CRC patient's specimens. This study investigated the prognostic significance of tumour CD26 expression in patients with CRC. Examination of CD26^+^ cells has significant clinical impact for the prediction of distant metastasis development in colorectal cancer, and could be used as a selection criterion for further therapy.

**Methods:**

Tumour CD26 expression levels were studied by immunohistochemistry using Formalin-fixed paraffin embedded (FFPE) tissues in 143 patients with CRC. Tumour CD26 expression levels were correlated with clinicopathological features of the CRC patients. The prognostic significance of tumour tissue CD26 expression levels was assessed by univariate and multivariate analyses.

**Result:**

CD26 expression levels in CRC patients with distant metastasis were significantly higher than those in non-metastatic. High expression levels of CD26 were significantly associated with advanced tumour staging. Patients with a high CD26 expression level had significantly worse overall survival than those with a lower level (p<0.001).

**Conclusions:**

The expression of CD26 was positively associated with clinicopathological correlation such as TNM staging, degree of differentiation and development of metastasis. A high CD26 expression level is a predictor of poor outcome after resection of CRC. CD26 may be a useful prognostic marker in patients with CRC.

## Introduction

Colorectal cancer (CRC) is the third most common malignancy and leading cause of cancer death in the world [1]. Approximately 50% of patients with CRC develop liver metastases, cancer patients with metastasis have higher mortality than just primary tumour alone [2], [3]. Liver is the most common site of distant metastasis in CRC patients with poor prognosis. Therefore, understanding the biological mechanism of metastasis is important for prognosis of metastasis in CRC.

CD26 – also known as dipeptidyl peptidase IV, is a 110-kDa cell surface glycoprotein protein with multiple functions, and is widely expressed in most cell types including T lymphocytes, endothelial and epithelial cells. It is a type II membrane-bound protein and member of prolyl peptidase family with carboxy terminus facing extracellular space. It is also composed of a transmembrane region and a short cytoplasmic domain. CD26 acts as other prolyl peptidase family: its carboxy terminal extracellular domain regulates the activities of a number of cytokines and chemokines through removal of the N-terminal dipeptide from polypeptides with proline or alanine in the penultimate position [4], [5]. CD26 can hydrolyse amino acid on matrix metalloproteinase [6]. CD26 was demonstrated as a binding partner with fibronectin and collagen in a variety of experimental conditions [7], [8], [9]. CD26 interacts with type I and III collagens and fibronectin, which proteolysis the ECM and result in facilitating the tumour cells migration, invasion and metastasis [10], [11], [12], [13]. Based on its ability to regulate biological molecules through its enzymatic activity, CD26 can act as a tumour suppressor or activator.

Our lab previously demonstrated that a subpopulation of CD26^+^ cells were associated with the development of distant metastasis in colorectal cancer through binding to extracellular matrix components such as fibronectin and collagen, and regulating the expression of EMT markers [13]. Besides its expression on tumour cell surface, the truncated form (sCD26/DPPIV) is also present in body fluids such as serum, and its levels are correlated with tumour status and behaviour for certain cancers. Serum CD26 levels were suggested as an early diagnosis and predictive marker of colorectal cancer [14]. Higher levels of circulating CD26 have been identified in CRC patients with metastatic disease [15]. All of these studies suggested that CD26 is a potential biomarker for CRC diagnosis and prognosis. The patient's specimens are another way to be used for detecting CD26 expression level by immunohistochemistry. Therefore, the aim of the present study was to clarify the prognostic significance of CD26 expression in patients with colorectal cancer.

### Patients and Methods

One hundred and forty three patients (81 men and 62 women; median age 73, range 29–92 years) who underwent resection of CRC at Queen Mary Hospital, The University of Hong Kong, between August 2008 and November 2011 were studied. No patient had received any preoperative treatment. The specimens were fixed with formalin and embedded with paraffin wax. The study was approved by Institutional Review Board of the University of Hong Kong/Hospital Authority Hong Kong West Cluster (HKU/HA HKW IRB), and the written informed consent was obtained from the patients for participation.

To evaluate the prognostic significance of CD26 expression in CRC patients, the association between CD26 expression and survival was studied. Five micrometres tissue sections were cut and antigen retrieval was achieved by boiling the sections in citric buffer, pH 8.0, in an oven for 10 mins. Adjacent normal colorectal tissues were used as controls, and negative controls were performed by replacing the CD26 antibody (Novus Biologicals, LLC) with phosphate buffered saline. Positive signals were evaluated by two independent assessors who were unaware of clinical outcomes, in five fields under light microscopy at a magnification of 400X. Staining intensity was scored as follows: 0-negative staining; 1-weak staining; 2-moderate staining and 3-strong staining.

All clinicopathological data and follow-up results were collected prospectively in a computerized database. Tumours were staged according to the pathological tumour node metastasis (TNM) classification. All patients were monitored every 3 months for detection of any recurrence or distant metastasis. The last patient in the cohort had been followed for 21 months.

### Statistical analysis

The χ^2^ test (or Fisher exact test where appropriate) was used for nominal variables. Survival rates were calculated by the Kaplan Meier method and compared between groups by the log rank test. Multivariate analysis of prognostic factors was performed by the Cox proportional hazard model. All statistical analyses were performed by SPSS 16 for Windows statistical software (SPSS, Chicago, IL). P value <0.05 was considered statistically significant.

## Results

**Figure 1:**
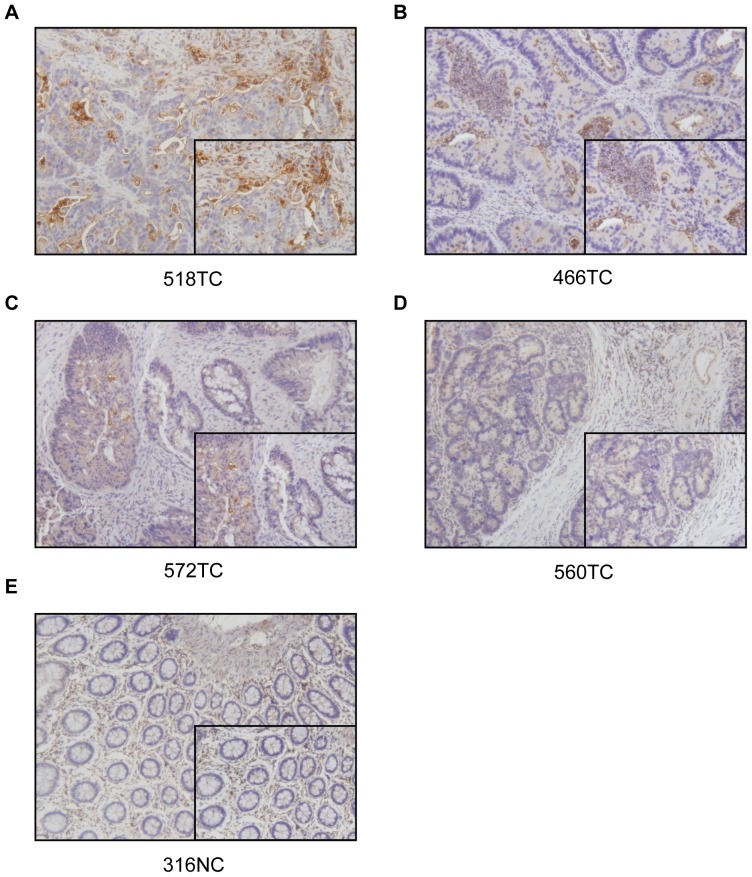
Immunostaining for CD26 on normal colon and CRC patient's specimen. (A-D) Colorectal cancer patient specimen with strong, moderate, weak and negative CD26 staining, respectively. (E) Normal colon. Magnification with 200x and 400x.

CD26 was expressed in CRC tumour specimens with different expression levels which were classified into four groups according to their staining intensity (strong, moderate, weak and negative staining) (Fig. 1). The CD26 expression level was further classified into high (staining intensity 2, 3) and low (staining intensity 0, 1) expression. The possible association between CD26 expression level and clinicopathological data was also examined (Table 1). There was no significant relationship observed between CD26 expression level and age, gender or tumour size. However, significantly higher CD26 expression was correlated with poorly differentiated tumour, late TNM stage, and presence of metastasis (especially liver metastasis). Moreover, CD26 expression was significantly associated with TNM stage III and stage IV (Table 2).

**Table 1:** CD26 expression categorized by clinicopathological data of CRC patients.

Variable		No. of cases^a^	CD26 Expression^a^		*P* *
			Low(n = 98) High(n = 45)		
Age (years old):	>65	42	24	18	0.075
	≤65	101	74	27	
Gender:	Male	81	56	25	0.858
	Female	62	42	20	
Tumour size (cm^3^):	>5	51	35	16	1.000
	≤5	92	63	29	
Degree of Differentiation:	Well and moderate	132	94	38	**0.029**
	Poor	7	2	5	
TNM stage:	Stage I/II	66	57	9	**<0.001**
	Stage III/IV	76	40	36	
Metastasis status^b^:	No metastasis	116	92	24	**<0.001**
	Metastasis	27	6	21	
Liver Metastasis:	No	123	92	31	**<0.001**
	Yes	20	6	14	

TNM, tumour node metastasis.

a The total number of cases may be less than 143 because of missing information.

b Patients with distant metastasis were classified as “Metastasis”.

*χ^2^ test (or Fisher exact test in 2-sided)

**Table 2:** CD26 expression categorized by TNM stage of CRC patients.

Variable	No. of cases^a^	Percentage	CD26 Expression^a^		*P* *
			Low High		
TNM stage (I, II and III, n = 115):					
Stage I/II	66	57%	57	9	0.037
Stage III	49	43%	34	15	
TNM stage (I, II and IV, n = 93):					
Stage I/II	66	71%	57	9	<0.001
Stage IV	27	29%	6	21	

TNM, tumour node metastasis.

a The total number of cases may be less than 143 because of missing information.

*χ^2^ test (or Fisher exact test in 2-sided)

**Figure 2:**
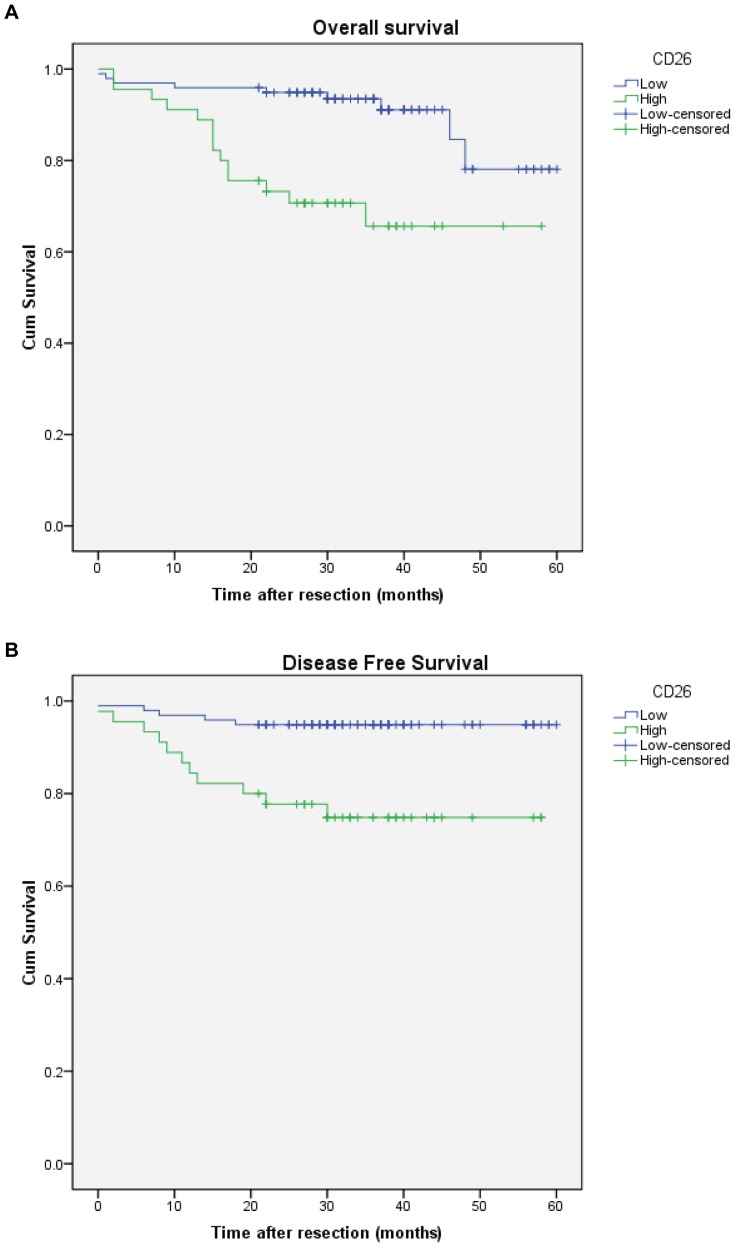
High CD26 expression associated with worse overall and disease free survival of colorectal cancer patients. A) Kaplan-Meier cumulative overall survival curves of CRC patients with high (expression level 3 and 2) and low CD26 expression. P<0.001 (log rank test). B) Kaplan-Meier disease free survival for CRC patients stratified by high and low CD26 expression. P = 0.001 (log rank test).

**Figure 3:**
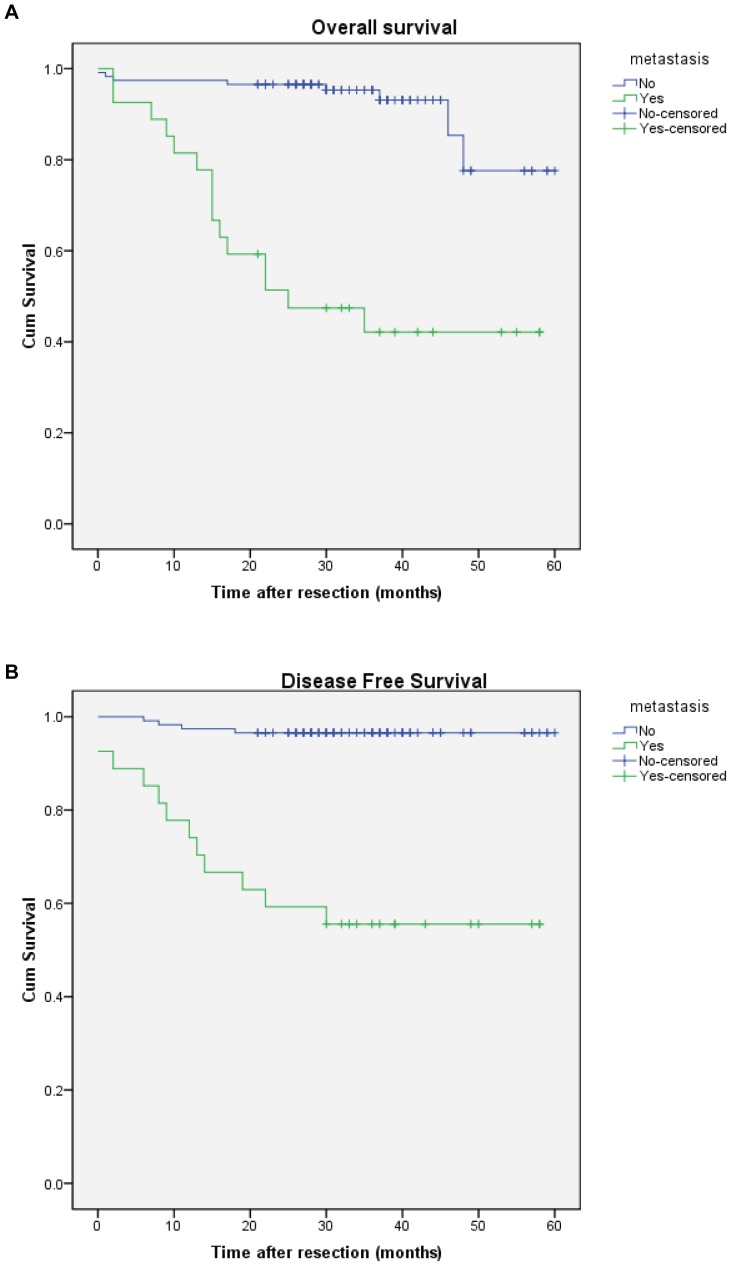
Metastasis associated with worse overall and disease free survival of colorectal cancer patients. A) Kaplan-Meier cumulative overall survival curves of CRC patients with metastasis and no metastasis. P<0.001 (log rank test). B) Kaplan-Meier disease free survival for CRC patients stratified by metastasis and no metastasis. P<0.001 (log rank test).

The median follow-up duration of the 143 patients with CRC was 32 months (range 0–60). The 1-, 3- and 5-year overall survival rates were 91, 66 and 66 per cent respectively in patients with high CD26 expression, and 96, 94 and 78 per cent respectively in those with low CD26 levels (*P*<0.001) (Fig. 2A). The survival curve of CRC patients included in this study is plotted in Figure 2B. The median disease-free survival time was 33 months (range 0–60) and the percentages of tumour recurrence were 25.2% and 5.1% for patients with high and low CD26 expression in five years, respectively. It means that the disease-free survival of patients with high CD26 expression was significantly worse than that of patients with low CD26 level (*P* = 0.001). Results shown in Figure 3 suggest that there was significant differences between patients with metastasis and those without metastasis in either overall survival (*P*<0.001) or disease-free survival (*P*<0.001). The percentages of tumour recurrence were 44.4% and 3.4% for patients with distant metastasis and without distant metastasis in five years, respectively.

Besides CD26 expression, table 3 shows the results of a univariate analysis of patient's clinicopathological features which might affect prognosis. From these parameters, only TNM stage, metastatic status and liver metastasis had significant long-term outcome in this study.

**Table 3:** Significant prognostic factors of overall survival by univariate analysis.

Variable	No. of cases	5-year survival (%)	*P* *
TNM stage:			
Stage I/II	64	92.4	0.021
Stage III/IV	78	77.6	
Metastasis status:			
No metastasis	116	93.1	<0.001
Metastasis	27	44.4	
Liver metastasis:			
No	123	90.2	<0.001
Yes	30	45	

*Log rank test

When CD26 expression (high versus low levels) was entered into a Cox regression analysis together with these three factors, only metastatic status was identified as independent prognostic factors of overall survival (Table 4A). However, CD26 expression level was an independent prognostic factor of overall survival when CD26 expression was entered into a Cox regression analysis together without metastatic status (Table 4B).

**Table 4:** Prognostic factors of overall survival by Cox regression analysis.

A: Variable	Hazard ratio	*P* *
TNM stage (I/II versus III/IV)	0.466 (0.089, 2.44)	0.366
CD26 expression (Low versus High)	1.953 (0.674, 5.656)	0.218
Metastasis status (No Metastasis versus Metastasis)	10.556 (1.731, 64.367)	0.011
Liver Metastasis (No versus Yes)	1.279 (0.392, 4.176)	0.683
B: Variable	Hazard ratio	*P* *
TNM stage (I/II versus III/IV)	1.029 (0.301, 3.514)	0.964
CD26 expression (Low versus High)	2.967 (1.122, 7.83)	0.028
Liver Metastasis (No versus Yes)	4.897 (1.772, 13.536)	0.002

Values in parentheses are 95% confidence intervals.

*Log rank test

**Figure 4:**
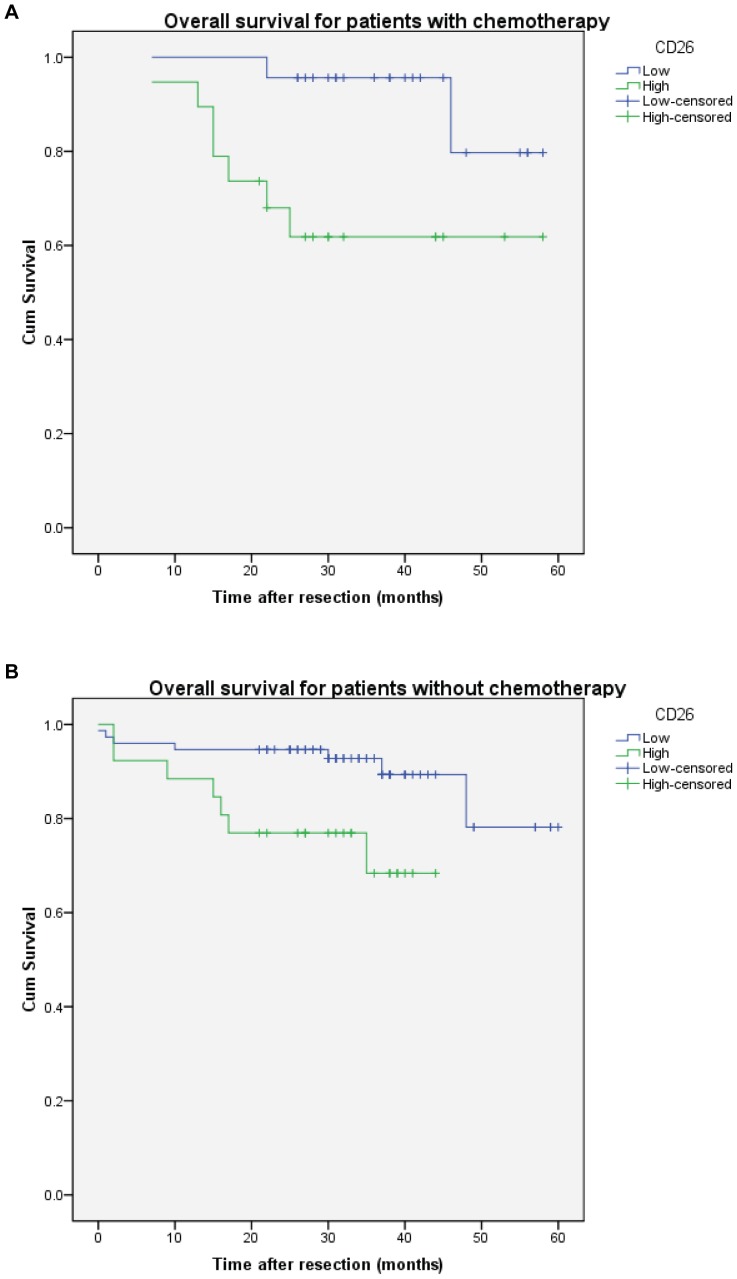
High CD26 expression associated with worse overall survival of colorectal cancer patients with or without chemotherapy. Kaplan-Meier cumulative overall survival curves of CRC patients with (A) and without (B) chemotherapy. P = 0.013 and 0.012 respectively (log rank test).

**Figure 5:**
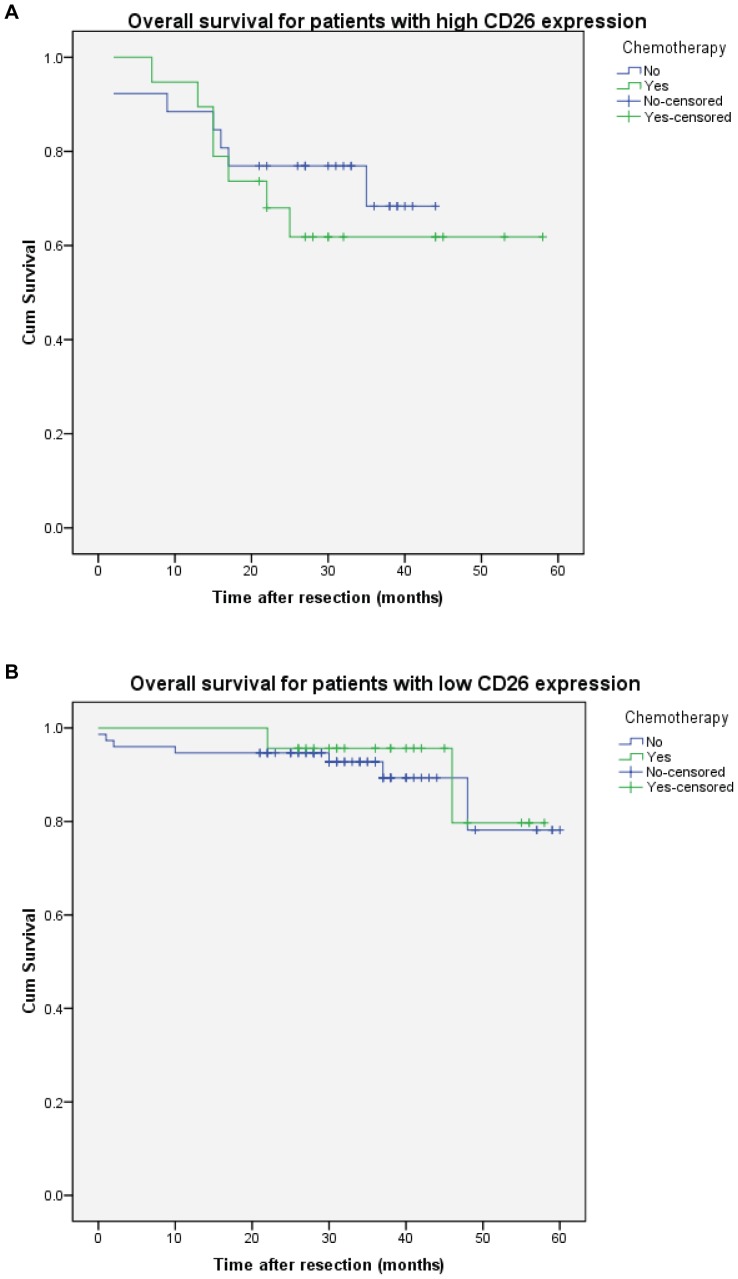
Chemotherapy has no effect on overall survival of colorectal cancer patients with high or low CD26 expression. Kaplan-Meier cumulative overall survival curves of CRC patients with high (A) or low (B) CD26 expression. P = 0.514 and 0.661 respectively (log rank test).

To study the response of chemotherapy, CRC patients with different CD26 expression were analysed with or without chemotherapy (5-Fu or oxaliplatin) (Fig. 4). The result showed that high CD26 expression was associated with worse overall survival whether with or without chemotherapy (P = 0.013 and 0.012 respectively). On the other hand, there was no significant effect of chemotherapy on CRC patients with high or low CD26 expression (Fig. 5). Figure 5A suggest that CRC patients with high CD26 expression seems have better survival for the first year with chemotherapy, but the survival became worse afterwards.

## Discussion

Colorectal cancer (CRC) is one of the most leading cause of cancer deaths in the world due to asymptomatic early stage and mostly diagnosis in advanced stages [1]. The 5 year survival rate of CRC patients with metastatic disease is less than 10% [16]. Colonoscopy and faecal occult blood test (FOBT) in stool are the two most common screening tool for CRC [17], [18], [19]. However, they have different disadvantages and limitations such as high cost for the population with low risk, discomfort of patients or low sensitivity [20]. Besides an asymptomatic early stage, metastasis is the major cause of high mortality in CRC. In order to predict the outcome of colorectal cancer accurately, there has been great interest to develop factors which can help for diagnosis or prognosis because survival can be dramatically improved at early detection and treatment of CRC [21].

CD26 is a multifunctional cell surface glycoprotein protein with intrinsic dipeptidyl peptidase IV (DPPIV) activity which widely expressed in most cell types. Cell surface proteases participate in cancer progression and malignant transformation by facilitating tumour cell invasion and metastasis [22]. Yamada K *et al*. recently demonstrated that anti-CD26 monoclonal antibody induced nuclear localization of CD26 from cell surface which can inhibit tumour cell growth [23]. Several studies have indicated that serum CD26 can be an early diagnostic marker for colorectal cancer [15], [24], [25]. However, they did not clarify whether CD26 expression had any independent prognostic significance. Moreover, the reports were conflicted in the concentration of soluble CD26 in CRC patients: one study found out that soluble CD26 was significantly higher in healthy donors, but another report showed higher level of soluble CD26 was detected in CRC patients. It varies among different papers, which may due to different detection methods in the study (one used ELISA assay and another one used an assay for enzyme activity).

Our previous study has clearly demonstrated that a subpopulation of cancer cells with CD26 expression were associated with the metastatic progression and chemoresistance of colorectal cancer [13]. Based on our previous findings, we further investigated the potential prognostic properties of CD26 expression on CRC patient's specimens.

In this study, our results showed that the high CD26 expression level was a significant predictor of both reduced overall survival (*P*<0.001, log rank test) and disease free survival (*P* = 0.001) in patients with CRC under univariate analysis, which means that higher CD26 expression has worse survival rate and higher rate of recurrence. Even though there is no correlation between CD26 expression and age, gender or tumour size, a significant difference in degree of differentiation (*P* = 0.029, chi-square test), TNM stage (*P*<0.001) or metastatic status (*P*<0.001) show that CD26 expression was positively associated with tumour differentiation, invasion and metastasis. It indicated that higher CD26 expression had poorer differentiation and higher potential for developing distant metastasis. Moreover, CD26 also has significant higher expression on TNM stage III (*P* = 0.037) or stage IV (*P*<0.001), which demonstrated that high expression of CD26 was associated with late TNM stage. In multivariate analysis, CD26 expression is a long-term survival independent pathological variable which have clinical implications when comparing with tumour stage (*P* = 0.028). It indicated that CD26 expression was a more significant prognostic marker than TNM stage. However, in the multivariate analysis including metastatic status, CD26 expression was not an independent predictor of overall survival which may due to dominant effect of metastatic status on patient survival.

CD26 has a function of binding to extracellular matrix proteins which may have a role in tumour migration and metastasis. Recent studies have demonstrated that CD26 binds to fibronectin and type 1 collagen, which are major components of the extracellular matrix, to facilitate the metastatic progression and invasive phenotype in CRC through down-regulation of E-cadherin [26], [27], [28]. Additionally, CD26+ cells were shown to possess greater cancer stem cell properties and chemoresistance when compared with CD26- cells [13]. Our previous findings also suggest that the CD26 protein in CD26+ cells does not only provide them with adhesion to both fibronectin and type I collagen but also give them EMT-like attributes, which contributes to the invasive phenotype and metastatic capacity of the CD26+ cells. It is consistent with our findings that CD26 expression is significantly associated with metastatic status (*P*<0.001). Taken together, it provides a potential explanation as to why higher CD26 expression is associated with poorer survival in CRC patients.

The new metastases is still frequently developed even the tumour response has been improved for systemic chemotherapy on CRC. There are more than 80% in situ tumour recurrences for CRC patients even after apparently complete radiological response to chemotherapy for liver metastasis [29]. As demonstrated in our previous in vitro and in vivo studies, CD26+ cancer stem cell subpopulation had enhanced chemoresistance [13]. Therefore, CRC patients with high CD26 expression may fail to eradicate the CD26+ cells under chemotherapeutic treatments which led to enrichment of CD26+ cells and ultimately cause further metastasis and worse survival after first year of treatment. Chemotherapy (5-Fu and oxaliplatin) didn't show improvement on CRC patients survival after surgical resection, it is crucial for development new therapeutic strategies targeting such CD26+ cancer stem cell.

Carcinoembrynoic antigen (CEA) is a tumour-associated antigen which was identified in CRC tissue in 1965 [30]. Patient's sera CEA level, who have tumours of digestive tract, was used as a most common diagnostic and prognostic marker for CRC [31], [32], [33], [34]. However, the postoperative CEA values were stabilized after 12^th^ post-operative week and the mean time of detectable rise in CEA for CRC patients is more than 3 months [35], which may lose the most suitable time for adjuvant therapy. On the contrary, the CD26 expression level can be detected after operation and the result can be used for prognosis.

In conclusion, our findings suggest that the specimen CD26 expression level is an independent prognostic marker which predicts survival significantly before development of metastasis, and it can provide additional prognostic information and allow selection of CRC patients at high risk of tumour recurrence or development metastasis for adjuvant or neoadjuvant therapy. CD26 expression may be a useful prognostic marker in patients with CRC after surgical resection.
